# Young adults’ sought gratifications from, and perceptions of food advertising by, social media influencers: a qualitative approach

**DOI:** 10.1186/s41043-023-00449-4

**Published:** 2023-09-26

**Authors:** Ghada Talat Alhothali, Najlaa M. Aljefree

**Affiliations:** 1 Marketing Department, College of Business, University of Jeddah, 3795 Jeddah, Saudi Arabia; 2 Food and Nutrition Department, Faculty of Human Sciences and Design, King Abdulaziz University, Jeddah, Saudi Arabia

**Keywords:** Social media, Influencers, Uses and gratifications, Young adults, Food advertising, Saudi Arabia

## Abstract

**Background:**

This study aims to explore young adults’ sought gratifications from social media influencers and whether they are exposed to food advertising by influencers. Further, it aims to understand how young individuals perceive food advertisements by social media influencers.

**Methods:**

This qualitative study was conducted on 17 students from two public universities—King Abdul Aziz and Jeddah universities—located in the City of Jeddah, Saudi Arabia, using in-depth, face-to-face, and semi-structured interviews. The participants were active users of social media platforms who followed at least one influencer. Thematic analysis was used to analyse the data. The COREQ guidelines for reporting qualitative research (see Additional file 2) were followed when stating the findings.

**Results:**

The findings reveal ten themes, that is, seven gratifications sought by young adults—broadening knowledge, perceived usefulness, self-improvement, boosting positivity, fostering morale, reinforcing inspiration, and passing time/enjoyment—and three themes (i.e. repeated, authentic, and unhealthy) that describe how Saudi young adults perceive food advertising.

**Conclusion:**

This study contributes to social media influencer marketing by identifying the factors that motivate young consumers to follow influencers, and it elucidates the extent to which young adults are exposed to food marketing, adding to the body of literature on food advertising.

**Supplementary Information:**

The online version contains supplementary material available at 10.1186/s41043-023-00449-4.

## Background

In recent years, businesses have shifted to advertising via social media influencers (SMIs), following the traditional advertising model where celebrities influence their fans strongly. Further, the unprecedented use of social media among populations has motivated companies to switch to social media advertising [1]. The popularity of social media usage among young consumers is understandable, as the generation has grown with the emergence of social media platforms. Prior studies have shown that digital food advertising is pervasive among young people of all ages, and people recall advertisements for unhealthy foods more frequently than they do advertisements for healthier options or for non-food products. In particular, Aljefree and Alhothali [1] studied Saudi Arabian young adults’ exposure to social media food advertising and found that 85.8%, 75.9%, 61.4%, 51.3%, and 50.3% of them use Snapchat, Instagram, YouTube, Twitter, and TikTok, respectively. Most importantly, the study found that obese participants were more likely than their non-obese counterparts to purchase foods or drinks after being exposed to food advertisements on social media.

Furthermore, a recent study has shown that young consumers are more likely to follow online influencers. In particular, 58% of young adults in the USA have purchased a product recommended by an online influencer [2]. Factors that motivate people to follow traditional media and, more recently, social media have been studied in the literature using theories such as the Uses and Gratifications (U&G) theory. Moreover, despite previous attempts to explore people’s gratifications from using social media (e.g., [3] and [4]), few studies have explored young adults’ motivation to follow SMIs [5]. In addition, social media platforms evolve with the motivations to use them [4]. Hence, this study fills these gaps. It uses the U&G theory as a guiding framework to explore the reasons that motivate young adults to follow SMIs. Using the U&G theory is justified as the topic regarding what motivates people’s media consumption remains vital, and it is always evolving in tandem with the media's dynamic change [4]. Hence, to fill this gap, this study has the following objectives:

1. To explore young adults’ gratifications from following SMIs
2. To explore whether they are exposed to food advertisements by SMIs
3. To investigate young adults’ perception of food advertisements by influencers

The rest of this paper is organised as follows: first, it presents an overview of the relevant literature that informed the study's aims and methods. Second, it explains the research design and analytical procedures thoroughly. Third, a comprehensive presentation of the outcomes is shown, followed by a discussion of the outcomes and concluding remarks.

### Literature review

#### Social media influencers

The Word-of-Mouth Marketing Association (WOMMA) defines influencer marketing as the process of defining key populations and opinion leaders who are likely to speak about goods and have the power to influence the opinions of others [6]. Social media celebrities [7] and influencers are not well known before their presence on social media. According to De Veirman et al. [7], they have created an online presence by disseminating information, creating content, and sharing aspects of their personal lives to keep followers engaged. The term micro-celebrity, or the ‘*condition of being famous to a narrow set of individuals*’, is commonly used to describe SMIs [8]. Furthermore, compared with traditional celebrities, SMIs are more likely to interact with their fans on a one-on-one basis. Particularly, they communicate with their followers by responding to their requests and incorporating their feedback into their content [9].

#### Uses and gratifications theory

The U&G theory has long been considered fundamental in interpreting users' behaviours in traditional media consumption. The key premise of the idea is that humans are goal-oriented and purposeful in their media use. Particularly, individuals are predisposed to selecting certain communication media from a vast selection to primarily satisfy a number of demands, including social and psychological ones [10]. Further, individuals’ satisfied needs are associated with their social role and psychological behaviour, often demonstrated by a reinforced or lessened connection to self, family, and community [10].

This theory’s inception dates back to the early 1940s, when it was utilised to explain the reasons behind people's selection of certain media. It has been widely used to interpret audience gratifications from traditional media such as listening to radio, watching TV, and reading newspapers [11]. The primary premises of the uses and gratifications framework were first articulated by Katz and colleagues in 1973. These include the following goals: to understand how people use media to meet their needs, to gain insight into the factors that motivate media consumption, and to determine the effects of these factors. Accordingly, uses and gratifications consider the following key points: ‘*(i) the social and psychological origins of (ii) needs, which generate (iii) expectations of (iv) the mass media or other sources, which lead to (v) differential patterns of media exposure (or engagement in other activities), resulting in (vi) need gratifications and (vii) other consequences, perhaps mostly unintended*’. [11]. Since then, numerous studies have employed the U&G theory to investigate how different types of media are used and appreciated by their audiences [3, 12]. Furthermore, the U&G theory has recently been deployed to investigate why people accept new technologies, particularly the internet and its associated media [12]. In particular, it has been used extensively to interpret people's acceptance of social media, such as Facebook [13], Instagram [14], Twitter [15], and Snapchat [16, 17]. The U&G theory is beneficial in interpreting the wide range of people’s motivation for engaging in social media use. Rooted in the communications field, the U&G theory can be applied to the social media field [3].

#### Motivations for following social media influencers

Whiting and Williams [3] presented pioneering research that identified the primary gratifications for using social media and found ten uses and gratifications: social interaction, information seeking and sharing, pass time, entertainment, relaxation, communicatory utility, convenience utility, self-expression, and surveillance/knowledge about others. Furthermore, prior studies have shown that individuals frequently follow celebrities and social influencers to learn more about a particular product or service [18]. In addition, Morton [5] investigated the factors that motivate young adults to follow SMIs and identified six motivations: information, inspiration, communication, entertainment, and surveillance. More specifically, Lee et al. [14] recently explored consumers’ motivations to follow influencers on Instagram and identified four motivations: authenticity, consumerism, creative inspiration, and envy. The study further tests the influence of these motivations on consumer behaviour outcomes such as trust and frequency of purchase [14]. Furthermore, Kircaburun et al. [19] investigated people’s differences and used motives in relation to problematic social media use, showing that younger people are significantly more inclined to use social media for entertainment purposes than for informational or instructional purposes. Further, young adults may have a wide range of reasons for following social influencers, from seeking and sharing information to seeking fun and friendship [20]. Particularly, previous studies have found that young adults are motivated to follow SMIs for entertainment, for instance, through humorous comedies [21] and to alleviate boredom [20].

Previous studies have demonstrated the influence of food advertisement on social media on young adults’ dietary behaviour [22, 23]. A few studies have explored the role of SMIs on young adults’ food consumption [24, 25]. However, limited studies have discovered young adults’ motivations to follow influencers on social media [5]. Hence, this study attempts to help understand young adults’ primary motivations to follow influencers on social media. The success of influencer marketing may depend on the specific sociopsychological wants and needs that consumers attempt to fulfil through their interaction with particular influencers; therefore, it is important to understand the motivational process involved in SMIs’ consumption. Of the numerous frameworks found in the literature, this study uses the framework of Whiting and Williams [3].

#### Perception of influencers’ advertisement and endorsement

Previous studies have shown that several factors influence followers’ perception of influencers’ marketing activities, such as followers’ attachment to the influencer, credibility and trust, and influencers characteristics [1, 26, 27]. Kim and Kim [9] conducted a study in the USA recently, finding that attachment increases followers' trust in and acceptance of endorsements but has no effect on their perception of the endorsements as advertisements. Furthermore, Konstantopoulou and colleagues [27] found that participants were sceptical about endorsements and recommendations from beauty influencers, as they did not always perceive them as genuine; they chose to conduct their own investigation. Their most fascinating discovery concerned the significance of trust, honesty, and authenticity in the influence of an e-Word of Mouth (e-WOM) [27].

Recently, businesses have been utilising digital endorsement via SMIs, which is a recent form of positive e-WOM, to support their presence online, increase their customer base and improve the effectiveness of advertisements [28, 29]. Particularly, an experimental study by Schouten et al. [28] shows that consumers are more likely to purchase products endorsed by an influencer rather than a celebrity. Compared to traditional celebrities, consumers perceive Instagram celebrities as more reliable, have a better opinion of the brands being promoted, and feel more connected to and jealous of them [30]. Influencers on social media appear informed and authoritative to their followers [31]. They equate their prominence and personal reputation with the social media content they endorse [31]. Moreover, scholars have considered the influencer characteristics’ role in influencing their followers’ behaviours [26]. By doing so, SMIs can affect how their followers perceive their product advertising by providing a welcoming and truthful atmosphere [32]. Furthermore, a growing body of literature discusses the impact of social media food advertisement on young adults’ dietary behaviours [1, 24, 33]. First, an investigation conducted by Coates et al. [24] revealed that young people who were exposed to SMIs promoting unhealthy meals consumed more calories and unhealthy foods than young people who were not exposed to such influencers. A recent study conducted on a sample of young children has shown that adolescents are exposed to social media food promotions that are mostly focused on unhealthy diets [25]. Furthermore, recent research has shown that young adults in Saudi Arabia are highly exposed to food commercials on social media platforms [1]. Although a growing body of literature has highlighted the role of food advertisements on social media followers’ behaviours, research on how followers perceive food advertisement by SMIs is scarce.

## Methods

Owing to the scarcity of research on the U&G theory and its relevance to the general phenomena of SMIs, particularly food advertisement, an exploratory investigation was carried out [3, 34].

The primary questions that the current study seeks to answer are: ‘What are the gratifications sought by young adults when following SMIs?’ ‘Are young adults exposed to food advertisements by SMIs?’ and ‘How do they perceive food advertisements?’.

As the goal of qualitative research is not necessarily to arrive at statistically valid conclusions, but to gain deeper insights and to enrich our understanding about the topic under investigation, it was selected as the primary method in this study. More specifically, self-report is useful in articulating reliable data regarding the expression of one’s motives, gratifications, and perceptions [11]. In-depth interviews were used to explore the meaning behind following SMIs. Literature review and consultation with experts informed the formulation of the interview questions (see Additional file 1).The interviews’ questions were pilot tested by conducting two interviews. No major changes to the questions were done. The two pilot interviews were among the 17 interviews. The interviews were conducted by the authors of this study.

This study used non-probability, purposive sampling to ensure that young students who were active users on social media platforms, and who followed at least one influencer, were recruited to participate. The students were recruited from the two public universities (i.e. King Abdul Aziz and Jeddah Universities) located in the City of Jeddah, Saudi Arabia. These universities were selected, as they have the largest number of students and they are the universities to which the authors belong.

### Data collection

The participants were either the authors’ students or the authors’ colleagues’ students. An invitation link was sent to students’ mobile numbers via WhatsApp. The link included an invitation to participate in the study, text describing the nature and purpose of the study, and a consent form. The authors shared the invitation link with colleagues to share with their students. Feedback was received when we first shared the invitation link with participants. Some respondents suggested dropping the question regarding how many people follow each influencer because they were unsure. Amendments to the questionnaire were done as recommended.

After getting their consent to participate, the participants were introduced to the study’s primary objectives and then asked to fill a short online questionnaire. The questionnaire had questions regarding the participants’ demographics and social media usage. Thereafter, the participants were asked to inform the researchers about the convenient time to conduct the interviews. The participants were informed that the interviews would be recorded.

The participants were not identified immediately, as the number of participants increased gradually as they agreed to participate. When the first round of interviews was conducted, the researcher encouraged the participants to send the interview link to their friends or colleagues who might be suitable participants in the study. No incentives were offered; however, participants will be informed of the findings once they are published. Seventeen interviews were completed— saturation was attained after reaching the seventeenth interview. The interviews ranged from 30 to 50 min. Both authors took field notes during the interview. Each interview was recorded and then transcribed verbatim. The interviews were conducted in Arabic, which was the interviewees’ preferred language. No follow-up interviews were conducted.

### Analysis

The research questions guided the analysis, which followed the procedures outlined by Miles and Huberman [35]. Considering the scarcity of high-quality qualitative analysis software that is in Arabic, the thematic analysis was conducted manually [29]. More specifically, we followed the steps of data reduction, data presentation, and conclusion verification in that sequence. Investigator triangulation was conducted to guarantee the relevance of responses, eliminate bias, and ensure content validity [36]. Investigator triangulation was utilised to strengthen the validity and reliability of the findings by having two researchers independently code and analyse the data and then compare the analyses [37].

Themes were reached by manually analysing the transcripts. Each interview was transcribed verbatim. Transcribing was first done in Arabic. Each author started the analysis by reading the transcripts for all participants many times. Each author generated codes and the corresponding quotes were organised into tables. Then, the authors shared the codes with each other and compare them. Similar codes were revised to ensure the same interpretation is attained. The authors then had a lengthy discussion about the identified themes to reach consensus on them. Some of the identified themes were eliminated as they were not relevant to the research questions. After the authors reached agreement, the codes, themes, and quotes were translated into English and back to Arabic to ensure consistency while making sure that the underlying meaning was preserved. Transcripts were not sent back to interviewees for review or editing.

Influencers' identities are coded as their initials, while participants' names are coded according to the institution they attend. The COREQ guidelines were followed when presenting the findings of this study (see Additional file 2).

## Results

The analysis of the socio-demographic data is presented in Table 1.

**Table 1:** Socio-demographic data of participants

Variables	Categories	Frequency	Percentage
Gender	Male	6	35.3
	Female	11	64.7
Age	18–21	8	47.1
	22–25	7	41.1
	26–29	2	11.8
Education	Graduate	3	17.6
	Undergraduate	14	82.4
Monthly income (SAR)	< 3000	2	11.8
	3000–7000	3	17.6
	7000–12,000	4	23.5
	12,000–20,000	3	17.6
	> 20,000	5	29.3

The analysis of the participants’ demographics, as displayed in Table 1, shows that more than half of the participants (64.7%) were female, and almost half of them were young, with 41% being in their early twenties. Young adults accounted for only 11.8% of all participants. Furthermore, most participants were undergraduates, and only 17.6% were graduates. Only 11.8% of the participants earned less than SAR 3000 per month, while 17.6% earned between SAR 3000 and SAR 7000 per month. Almost a quarter of the participants made more than SAR 7000, and 17.6% earned between SAR 12,000 and SAR 20,000; however, more than a quarter of them made over SAR 20,000. The importance of the students’ monthly salary as an indicator of their purchasing power cannot be overstated. Even though over a quarter of them had annual incomes of less than SAR 7000, they were prompted to purchase owing to the impact of SMIs.

Table 2 displays the characteristics of young adults’ usage of social media. Almost half of the participants followed between one and five influencers. Approximately 30% of the participants followed six to ten influencers. The majority of the interviewed participants followed content creators' influencers. Snapchat was used by the vast majority of the participants, and 17.6% of them were using Snapchat and other social media platforms such as Twitter, YouTube, and Instagram. Instagram was used by approximately 6% of the participants. Almost half of the participants were following mega influencers (i.e. those with more than 1 million followers).

**Table 2:** Characteristics of social media usage

Participant #	Range of influencers	Field of fame	Platform
UJ1	[11–16]	Content creators	Snapchat
UJ2	[1–5]	Make-up artists	Snapchat
UJ3	[6–10]	Content creators	Snapchat
UJ4	[11–16]	Athletes	Snapchat
UJ5	[17–20]	Content creators	Snapchat
UJ6	[6–10]	Content creators	Snapchat
UJ7	[1–5]	Chefs; content creators	Snapchat
UJ8	[6–10]	Content creators	Snapchat; TikTok
KAU9	> 50	Content creators	Snapchat and Twitter
KAU10	[1–5]	Athletes	Snapchat
KAU11	[1–5]	Content creators	Snapchat
KAU12	[1–5]	Content creators	Snapchat
KAU13	[6–10]	Athletes Content creators	Snapchat YouTube Instagram Twitter
KAU14	[1–5]	Chefs	Snapchat
KAU15	[6–10]	Content creators	Snapchat
KAU16	[1–5]	Content creators	Snapchat
KAU17	[1–5]	Chefs	Instagram

King AbdulAziz University participants are coded (KAU#); University of Jeddah participants are coded (UJ#)

### Sought gratifications

Table 3 presents the primary themes and subthemes and the relevant interview excerpts.

**Table 3:** Thematic coding and proof quotes for sought gratifications

Theme	Sub-theme	Interview excerpts
Broadening knowledge	Broaden knowledge	I follow others because they are specialists and I find out more about their field. They share specific knowledge about a specific field of science (e.g. HR and logistics specialist). For me, they seem to be useful as they share practical and up-to-date information
	Relevant information	As a make-up artist, I follow influencers who provide specialised make-up application and beautification techniques
Perceived Usefulness	Useful techniques for daily routine Relevant content Useful content	For instance, the first influencer, FH, focuses on motherhood. She teaches followers how to schedule the meals for her baby. She also focuses on techniques that simplify her role as a mother and how to behave in certain situations
Self-improvement	Reach a goal	I enjoy following sports influencers, celebrities, and make-up artists. I follow sports and athlete accounts in order to learn how to train when I visit the gym. I need to lose weight so that I follow them to reach my goal. I observe them and learn more about how to burn calories and build my muscles via practice. They also advertise healthy food and stuff I follow one of the superstars, SS, because of his muscular appearance. His appearance is reflected in his daily routine. Despite eating a lot, he also spends a lot of time at the gym to burn off the calories. When I go to the gym, I recall that he previously showed us how to use this equipment, so I practice similarly without being injured
	Practical knowledge	
	Developing Skills	
Self-improvement	Thoughts provoking Diverse point of views Openness to others	The content MS shares is fantastic. His stories are focused on helping others improve themselves and achieve their goals in all areas of life. Every day, I look forward to seeing what he has posted on his account. His work is accessible, entertaining, and thought provoking. It's a great way to broaden your horizons and gain exposure to new insights and perspectives
Boosting positivity	Positive energy Fulfilling life style Positive attitude Positive aspects of life Full of life	I find that following some influencers gives me a boost of enthusiasm and positive energy, so I seek out more like them. YS is a great example of a role model because she encourages her followers to have a positive attitude, and she tries to set a good example by living a happy, fulfilling life herself. I learn how to maintain a healthy and fulfilling lifestyle by imitating those I admire I also follow other accounts that share social content and they have a positive impact on me. Each one of them is specialised in a certain positive aspect of life
Fostering morale	Good manner Considerate way Values Ethics	A third demonstrated her routine in a dignified and considerate way. She promotes ethical concepts, values, and morals and encourages the best in others
Reinforce Inspiration	Motivated Inspired Positive energy Accomplishment Balance	I believe their material boosts morale through positive energy, accomplishment, and inspiration. When I see how they balance their home responsibilities and work, I become more motivated. Their daily lives can show us young people what to do as well as how to behave in the future, when we have similar responsibilities
Passing time/Enjoyment	Boredom Spend time Enjoy Common interests Funny	Actually, I follow some because I sometimes feel bored I follow Snapchat influencers to spend time and for fun; however, Instagram is more formal and education- oriented As someone who finds enjoyment in the kitchen, I also follow an influencer who frequently posts recipes and tips on how to improve one's cooking” All these influencers are able to share information by displaying their real aspects of life in a funny and enjoyable way”

The themes presented in Table 3 represent the gratifications sought by the respondents.

When attempting to answer the first research question, ‘What are the gratifications sought by young adults when following SMIs?’, seven themes were salient in the data: broadening knowledge, perceived usefulness, self-improvement, boosting positivity, fostering morale, reinforcing inspiration, and passing time/enjoyment. ‘Broadening knowledge’ refers to the utility of following influencers to expand practical knowledge, whereas ‘perceived usefulness’ is related to the utility of observing influencers’ daily routines to acquire new skills. Furthermore, ‘self-improvement’ concerns the belief that following SMIs helps individuals to improve themselves and reach their objectives, whereas ‘boosting positivity’ refers to the fact that following SMIs improves individuals’ mood and sense of well-being. Moreover, ‘fostering morale’ is primarily related to the positive role of SMIs in encouraging morale and good deeds, and ‘reinforcing inspiration’ is largely concerned with SMIs' positive role in promoting inspiration. Finally, ‘passing time/enjoyment’ relates to a youngster’s need to spend time in enjoyment.

Three more themes were identified when the respondents were asked about food advertisements. When participants were asked whether they are exposed to food advertising through SMIs, 100% of them responded yes. Furthermore, when they were asked how they perceive SMIs' food advertising, various perspectives emerged in the data (Table 4).

**Table 4:** Thematic coding and proof quotes for respondents’ perception of commercials

Theme	Codes	Interview excerpts
Repetition	Boring Annoying Skipping the ads	Yes, I am exposed to plenty of advertising. If I am interested in the product, I continue watching; if not, I usually skip the ads. Repeated ads annoy me, so I usually don't even look at the account till the end of the day
Authentic	Genuine True Appealing High quality good Show-off Fake	One of the influencers is someone I adore and trust a lot. The fact that I've followed her suggestions before and been pleased with the results has given me reason to trust her. The advice she gives me is automatically accepted as good. In my opinion, what she said was correct. All of what she stated became associated in my head with being true. Honestly, she affected me in all aspects of my life I think that the food that influencers promote is high quality, appealing, and tempting, which makes me more likely to buy it Most of the commercials focus on the aesthetics of the meal (i.e. meal presentation and packaging), not the quality or taste. People nowadays are looking for a nice dish to photograph and share with friends People nowadays purchase more than they need to demonstrate their status. All of this is attributable to influencer marketing Most food commercials are fake. There is a disparity between the advertisement and the flavour of the food
Unhealthy	Fast food Deserts Carbs Gaining weight	In the past, I used to follow an influencer whose material was focused on unhealthy food advertisements, but I stopped doing so because I was gaining a significant amount of weight at the time. In order to lose weight, I quit following these influencers. Recently, I began to follow an influencer whose content is informative, such as historical stories. Typically, he mixes commercial goods into his content, such as dates and honey

Respondents believe that food advertisements by SMIs were repeated, authentic, and unhealthy. ‘Repeated’ refers to the frequency of showing the advertisements on the influencers’ accounts; ‘authentic’ refers to the credibility for the advertised products; and ‘unhealthy’ refers to food that is described as fast and full of carbs, sugar, and high calories.

### Discussion

#### Broadening knowledge

When participants were asked about the gratifications sought when following SMIs, 36% responded that they wanted to learn new things. Specifically, they wanted to broaden their practical knowledge in areas such as human resources, marketing, cooking, and training, among others. As presented in Table 3, a student, UJ2, who is interested in beauty, affirmed that the primary reason behind following SMIs is to learn practical techniques: ‘*As a make-up artist, I follow influencers that provide specialised make-up application and beautification techniques’.* Another participant, UJ4, confirms that the gratification sought is learning how to practice at the gym: ‘*I follow one of the superstars, SS, because of his muscular appearance. His appearance is reflected in his daily routine. Despite eating a lot, he also spends a lot of time at the gym to burn off the calories. When I go to the gym, I recall that he previously showed us how to use this equipment, so I practice similarly without being injured’.* This theme affirms ‘*information seeking*’, which was identified by Papacharissi and Rubin [38] and Whiting and Williams [3] when attempting to explore the uses and gratifications of using the internet and social media, respectively. Moreover, these findings corroborate previous studies showing that young adults seek out influencers who can deliver life-relevant, practical information [39].

#### Perceived usefulness

The value of following SMIs to discover new skills and observe how other people live is tied to this theme. Influencers on social media engage with their audiences by posting about their daily routines and other original content. The majority of this study’s participants stress that most influencers deliver valuable information. One of the respondents, UJ5, claims that one influencer shares relevant and useful information, ‘*Like myself, she is currently enrolled in college, and I enjoy following her to gain insights into the everyday routines of other undergrads. She also shares useful apps’.* UJ6 stressed that most of the influencers she followed share social content that is useful and informative. ‘*FH, focuses on motherhood. She teaches followers how to schedule the meals for her baby. She also focuses on techniques that simplify her role as a mother and how to behave in certain situations’.* This finding confirms a previous study by Barbe et al. [40], which found the primary motivation for following travel influencers as usefulness, enjoyment, and entertainment. Based on the above results, we argue that SMIs have succeeded in attracting young people by sharing with them their appealing daily routines and lifestyles. Furthermore, the content they share with their fans has satisfied their curiosity to learn about new lifestyles and different backgrounds and cultures, and to have fun.

#### Self-improvement

UJ6 affirms that one of the influencers fosters followers to achieve their goals, ‘*The content MA shares is fantastic. His stories are focused on helping others improve themselves and achieve their goals in all areas of life. Every day, I look forward to seeing what he has posted on his account’.* She further explains how the influencer is helping the followers to widen their knowledge and perspectives: ‘*His content is valuable and rich in providing self-development information’.* UJ6 further elaborates how deep the information is that he provides to improve people's thoughts, beliefs, and attitudes on life's challenges and how’*his work is approachable, engaging, and thought provoking. It's a great way to broaden your horizons and gain exposure to new insights and perspectives’.* Another participant, UJ4, confirmed this by stating, ‘*I follow sports and athlete influencers to learn how to train when I visit the gym. I need to lose weight, so I follow them to reach my goal’.* This result confirms the findings of the study by Malik et al. [41] which found that influencers satisfy their followers’ need to improve themselves. Improving oneself is another gratification sought by youngsters from following influencers. Hence, we believe that influencers have succeeded in positively impacting their followers to achieve their goals and perform the desired actions. This result contradicts the previous literature about the detrimental impact of social media on young people, as we argue that if young adults choose the right social media figure to follow, they will reap the resulting benefits.

#### Boosting positivity

Participants stress that following successful people on social media boosts their energy and stimulates positivity. Respondents confirm this concept by mentioning *‘enthusiasm’*, ‘*boosting energy*’, and ‘*fulfilling happy life*’. UJ4 describes her experience with one of her favourite influencers by saying ‘*I find that following some influencers gives me a boost of enthusiasm and positive energy, so I seek out more like them. YA is a great example of a role model because she encourages her followers to have a positive attitude, and she tries to set a good example by living a happy, fulfilling life herself. I learn how to maintain a healthy and fulfilling lifestyle by imitating those I admire’.* These results confirm previous studies into the impact of role models, which are viewed as a tool to motivate individuals to engage in innovative behaviours and encourage them to establish high ideals [42, 43].

Furthermore, UJ6 claimed that watching the influencer’s content makes her feel better, ‘*Imaging her little children, full of life and enthusiasm, brightened my day’.* She further stresses that, ‘*each one of these stories has reminded me of a positive aspect of our existence. I feel better when I see their content’.* Accordingly, this theme adds to the positive influence of following SMIs on youngsters’ well-being and positive feelings. This finding contradicts prior studies, which demonstrate the problematic uses of social media [19].

#### Fostering morale

Respondents affirmed the role of influencers and their content in fostering good manners and values among their followers. UJ6 accentuates that ‘*an influencer shares her routine in a dignified and considerate way. She promotes ethical concepts, values, and morals and encourages the best in others’.* This finding supports previous studies on the role of influencers in influencing attitudes and behaviours such as wearing the hijab [44]. Based on these findings, we conclude that SMIs have a major effect on young people’s beliefs and actions.

#### Reinforcing inspiration

UJ6 emphasises the role of influencers in the lives of aspiring young people through valuable and inspiring content. UJ6 further elaborates, ‘*I believe their material boosts morale through positive energy, accomplishment, and inspiration. When I see how they balance their home responsibilities and work, I become more motivated. Their daily lives can show us young people what to do and how to behave in the future when we have similar responsibilities’.* Using external stimuli to inspire customers is not new to the marketing discipline. Prior research has studied the meaning, factors, and outcomes of customer inspirations [45]. As a result, our study findings support prior studies stating that customers are increasingly looking for sources of inspiration to help them achieve their ideal selves and their desired lives [46]. This finding reinforces the previous themes about the significant roles of influencers in attracting, satisfying, and inspiring followers.

#### Passing time/enjoyment

As the results indicate, most respondents were motivated by a desire to pass time and enjoy themselves. A participant reinforced this need by saying, ‘*I follow Snapchat influencers to spend time and for fun; however, Instagram is more formal and education-oriented’.* Another one affirmed that, ‘*All these influencers are able to share information by displaying their real aspects of life in a funny and enjoyable way’.* These results also corroborate previous studies that show how the need for entertainment and spending time is prevalent among youngsters [20, 21].

### Respondents’ perception of influencers’ food advertisement

Each of the emerged themes is discussed as follows:

#### Repetition

A subset of respondents shared this theme, indicating that they found commercials for products, including food, to be repetitive. In particular, commercials for non-food products were repetitive, annoying, and tiresome. Participants typically ignore and avoid this type of advertisements. One of the respondents confirms this by saying, ‘*Repeated ads annoy me, so I usually don't even look at the account till the end of the day’.* This supports previous research that found that with increased exposure to advertisements, a negative reaction takes over and the stimulus effect decreases [47]. However, people perceive food commercials as useful and enjoying, despite their repetition. A participant confirmed this finding by emphasising, ‘*Yes, I see food commercials. Most of them are fast food. One of the influencers repeat a commercial of a restaurant that she owns and the repetition encourages me to think of visiting her restaurant’.* These results support previous studies that show the significant influence of TV, food advertisements, and eating behaviour [48]. This result also corroborates previous research which shows that young consumers perceive fast food ads on social media as useful and entertaining [49].

#### Authentic

The vast majority of participants perceived food advertisements made by SMIs as genuine and enjoyable. In particular, they believed that the food was actually as advertised. For instance, UJ10 states that, ‘*One of the influencers is someone I adore and trust a lot. The fact that I've followed her suggestions before and been pleased with the results has given me reason to trust her. The advice she gives me is automatically accepted as good. In my opinion, what she said was correct. All of what she stated became associated in my head with being true. Honestly, she affected me in all aspects of my life’.* KAU9 agreed with UJ10 by saying, ‘*Yes, this month I have seen a burger commercial with pomegranate which is unusual. Then, I became excited to try this new product, which I tried, and was good’.* KAU12 who shared the same idea said, ‘*I think that the food that influencers promote is high quality, appealing, and tempting, which makes me more likely to buy it’.* This finding also corroborates a previous study by Gaber and Wright [49], which found that consumers perceive fast-food advertisements on Facebook as trustworthy and entertaining.

However, a minor group of respondents perceived food advertisements by SMIs as unauthentic representations of the products being promoted. According to KAU10, the ads and the product differ. He then reaffirmed, ‘*Most food commercials are fake. There is a disparity between the advertisement and the flavour of the food’.* In agreement with KAU10, UJ7 emphasised that food commercials recently are concerned with a superficial lifestyle, saying, ‘*Most of the commercials focus on the aesthetics of the meal (i.e. meal presentation and packaging), not the quality or taste. People nowadays are looking for a nice dish to photograph and share with friends’.* This finding lends credence to previous studies that found that young adults shared food-related images online to promote a desired lifestyle [50].

#### Unhealthy

This theme is related to the participants’ perception of food commercials by influencers on social media. The majority of respondents agree that they are exposed to numerous food commercials. Most of them emphasise that the food commercials they are exposed to advertise ‘unhealthy food’, ‘fast food’, ‘Carbohydrates’, and ‘sweets’. Additionally, when they were asked about whether the food advertised by SMIs is healthy or unhealthy, the majority confirmed that it is unhealthy: ‘*Yes, I also view plenty of food and restaurants commercials. Most of the advertised foods are unhealthy. None of the influencers I follow advertise healthy stuff’.* Another participant responded by saying, ‘*Yes, I see plenty of commercial products. Most of the advertised foods are Carbs*’, and the rest of the participants stated that they were exposed to both types (i.e. healthy and unhealthy). UJ8 further emphasised that, ‘*70% of advertisements are for unhealthy food, whereas influencers whose content promotes a healthy lifestyle promote healthy products*’. Another respondent emphasised that following influencers promoting unhealthy food led him to gain weight, he further states, ‘*In the past, I used to follow an influencer whose material was focused on unhealthy food advertisements, but I stopped doing so because I was gaining a significant amount of weight at the time. In order to lose weight, I quit following these influencers. Recently, I began to follow an influencer whose content is informative, such as historical stories. Typically, he mixes commercial goods into his content, such as dates and honey’.* These results support previous research on young adults' exposure to unhealthy food commercials [33, 50]. According to Qutteina et al. [33], 67% of the sampled adolescents are exposed to non-core food commercials on social media (i.e. platforms, influencers, peers) such as sweets, drinks, and pizza, among others.

### Limitations

The current study has a few limitations that must be considered. The participants were young adults in a single city in Saudi Arabia; thus, more research is needed to generalise the findings. Further, the study only considered how often young individuals saw food advertisements by SMIs; therefore, more research is needed to see how that exposure affects their feelings, decisions, and actions. Finally, as this study was done before the Disclosure Law was enacted in Saudi Arabia, more research should be conducted to determine how young adults’ views on food advertising have changed following its implementation.

## Conclusions

The phenomenon of SMIs is coupled with the massive adoption of social media platforms by young adults and a massive increase in food commercials by influencers. Therefore, this study investigates the gratifications sought by young adults from SMIs, as well as whether the influencers expose them to food advertisements and how they perceive these commercials. The study’s findings emphasise the U&G theory’s effectiveness in illuminating the factors that initially entice young adults to follow SMIs. This research contributes to social media influencer marketing by identifying the factors that motivate young consumers to follow influencers. Furthermore, the findings shed light on the extent to which young adults are exposed to food marketing and how they interpret food ads, adding to the body of literature on food advertising.

The results of this research enrich our understanding of why youngsters follow SMIs and to what extent the influencers impact young adults’ lives. This research has found that SMIs have a considerable impact on youngsters feelings, attitudes, and behaviours. This implies that SMIs represent a double-edged weapon. This means that if influencers deliver positive, inspiring, and useful content, youngsters will reap the benefits of this content, and vice versa. Hence, policymakers should control SMIs' content and ensure that the delivered content is positive, energetic, and inspiring. More policies should be implemented to mitigate the negative influence of the influencers’ content on young adults. Furthermore, social media influencers should be encouraged to foster morale, good deeds, and positivity among their followers and to be good role models for them. As part of their social responsibility, SMIs should also use their content to disseminate knowledge about social well-being. For instance, by creating content related to the negative impact of smoking and drugs.

### Managerial implications

This research elucidates how marketing managers may make the most of SMIs to further the corporate social responsibility initiatives of their companies. The government and public health policymakers in Saudi Arabia should limit the ability of SMIs to advertise unhealthy food products. This is because governments determine the overall direction of public health strategies and the scope of marketing restrictions in their respective countries [51]. Given the profound impact of SMIs on the diet and shopping habits of young adults, it is imperative that they be prohibited from engaging in unhealthy food advertising.

### Supplementary Information

**Additional file 1**. Interview questions.

**Additional file 2**. COREQ quidelines.

## Data Availability

None of the datasets generated and/or analysed in this study are publicly available because the authors require them for further publication. However, the data are available from the corresponding author upon request.

## Abbreviations

SMI: Social media influencer
U&G theory: Uses and gratifications theory
WOMMA: Word-of-Mouth Marketing Association
e-WOM: E-Word of Mouth
