# Supplementary material for: Young adults’ sought gratifications from, and perceptions of food advertising by, social media influencers: a qualitative approach
Source: J Health Popul Nutr. 2023 Sep 26;42:103. doi: 10.1186/s41043-023-00449-4 (PMC10521410; doi:10.1186/s41043-023-00449-4)
Supplement: Supplementary file 1 — Additional file 1. Interview questions. [file 41043_2023_449_MOESM1_ESM.docx]

**Interview questions:**

| Topic | Main questions | Follow up questions |
| --- | --- | --- |
| Social media influencer | Do you follow social media influencers? | If yes, how many? What are they famous for?  On which social media applications:  Snapchat  TikTok  YouTube  Twitter  Instagram |
| Motivations | Why do you follow social media influencers?  On what basis do you choose to follow them? |  |
| Exposure | Are you exposed to social media influencers advertisements?  Are you exposed to social media influencers’ food advertisements?  Do you remember having seen any of the social media influencers’ advertisements? |  |
