# Supplementary material for: Young adults’ sought gratifications from, and perceptions of food advertising by, social media influencers: a qualitative approach
Source: J Health Popul Nutr. 2023 Sep 26;42:103. doi: 10.1186/s41043-023-00449-4 (PMC10521410; doi:10.1186/s41043-023-00449-4)
Supplement: Supplementary file 2 — Additional file 2. COREQ quidelines. [file 41043_2023_449_MOESM2_ESM.docx]

**COREQ guidelines**

|  | **Guide questions/description** | **Remarks** | **Page no.** |
| --- | --- | --- | --- |
| Domain 1: Research team and reflexivity | | |  |
| *a*). *Personal Characteristics* | | |  |
| 1. Interviewer/ facilitator | Which author/s conducted the interview or focus group? | Two authors (GTA and NA) conducted the in-depth interviews. | 8 |
| 2. Credentials | What were the researcher’s credentials? *E.g*. PhD, MD | The researchers’ credentials are as follows:  GTA: PhD  NA: PhD | N/A |
| 3. Occupation | What was their occupation at the time of the study? | The researchers’ occupations are as follows:  GTA: academic  NA: academic | 1 |
| 4. Gender | Was the researcher male or female? | The researchers’ gender are as follows:  GTA: female  NA: female | N/A |
| 5. Experience and training | What experience or training did the researcher have? | Both GTA and NA have extensive backgrounds in qualitative research and have published numerous articles on the topic. | N/A |
| *b*). *Relationship with participants* | | |  |
| 6. Relationship established | Was a relationship established prior to study commencement? | Only for the purposes of this research. |  |
| 7. Participant knowledge of the interviewer | What did the participants know about the researcher? *e.g*. personal goals, reasons for doing the research | Both authors are well-known academics at the university where the participants studied. Everyone involved in the interview was aware, however, that it was being conducted for academic purposes. | N/A |
| 8. Interviewer characteristics | What characteristics were reported about the interviewer/facilitator? *e.g*. Bias, assumptions, reasons and interests in the research topic | None of the characteristics of each author have been reported in the paper. | N/A |
| Domain 2: study design | | |  |
| *a*). *Theoretical framework* | | |  |
| 9. Methodological orientation and Theory | What methodological orientation was stated to underpin the study? *e.g*. grounded theory, discourse analysis etc | Thematic analysis as utilised to analyse the data. | 1 |
| *b*). *Participant selection* | | |  |
| 11. Method of approach | How were participants approached? *e.g*. face-to-face, telephone, mail, email | An invitation link was sent to students’ mobile numbers via WhatsApp. | 8 |
| 12. Sample size | How many participants were in the study? | 17 students participated in the study. | 9 |
| 13. Non-participation | How many people refused to participate or dropped out? Reasons? | None of the approached participants has refused to participate. | N/A |
| *c*). *Setting* | | |  |
| 14. Setting of data collection | Where was the data collected? *e.g*. home, clinic, workplace | Data were collected at the authors workplaces. | N/A |
| 15. Presence of non-participants | Was anyone else present besides the participants and researchers? | Participants and researchers were the only ones present. | N/A |
| 16. Description of sample | What are the important characteristics of the sample?  *e.g*. demographic data, date | Important sample characteristics included age, gender, educational background, and monthly income. Further characteristics about the sample social media usage were also provided in (Table 2). | 10 and 29 |
| *d*). *Data collection* | | |  |
| 17. Interview guide | Were questions, prompts, guides provided by the authors? Was it pilot tested? | An interview guide was prepared (Additional file 1) and pilot-tested with two participants. In-depth interviews done during the pilot were incorporated into the final data set. | 8 |
| 18. Repeat interviews | Were repeat interviews carried out? If yes, how many? | No follow-up interviews were conducted. | 9 |
| 19. Audio/visual recording | Did the research use audio or visual recording to collect the data? | Interviews were audio recorded. | 9 |
| 20. Field notes | Were field notes made during and/or after the interview or focus group? | Both authors took field notes during the interview. These field notes supplemented the interpretation of the recorded interviews. | 9 |
| 21. Duration | What was the duration of the interviews or focus group? | The interviews ranged from 30 to 50 minutes | 9 |
| 22. Data saturation | Was data saturation discussed? | Data saturation was noted in data collection section | 9 |
| 23. Transcripts returned | Were transcripts returned to participants for comment and/or correction? | Transcripts were not sent back to interviewees for review or editing. | 10 |
| Domain 3: analysis and findings | | |  |
| *a*). *Data analysis* | | |  |
| 24. Number of data coders | How many data coders coded the data? | Two authors coded the data and worked separately. | 9 |
| 25. Description of the coding tree | Did authors provide a description of the coding tree? | Each author started the analysis by reading the transcripts for all participants many times. Each author generated codes and the corresponding quotes were organized into tables. Then the authors shared the codes with each other and compare them. Similar codes were revised to ensure the same interpretation is attained. The authors then had a lengthy discussion about the identified themes to reach consensus on them. Some of the identified themes were eliminated as they were not relevant to the research questions. After the authors reached agreement, the codes, themes, and quotes were translated into English and back to Arabic to ensure consistency while making sure that the underlying meaning was preserved | 9-10 |
| 26. Derivation of themes | Were themes identified in advance or derived from the data | The themes were derived from the data during analysis. | 10 |
| 27. Software | What software, if applicable, was used to manage the data? | The analysis was done manually. | 10 |
| 28. Participant checking | Did participants provide feedback on the findings? | The results were not discussed with the participants. | N/A |
| *b*). *Reporting* | | |  |
| 29. Quotations presented | Were participant quotations presented to illustrate the themes / findings?  Was each quotation identified? *e.g*. participant number | Yes, the quotations were provided, and the respondent number and the universities' initials were included. | See for example page 13 |
| 30. Data and findings consistent | Was there consistency between the data presented and the findings? | Yes, there was agreement between the data and the results. | N/A |
| 31. Clarity of major themes | Were major themes clearly presented in the findings? | Yes, major themes was clearly presented in the findings. | 12 |
| 32. Clarity of minor themes | Is there a description of diverse cases or discussion of minor themes? | No, there was no descriptions of minor themes. | N/A |
